# Pathophysiology of the Neutropenia of GSDIb and G6PC3 Deficiency: Origin, Metabolism and Elimination of 1,5‐Anhydroglucitol

**DOI:** 10.1002/jimd.70085

**Published:** 2025-09-16

**Authors:** Maria Veiga‐da‐Cunha, Lila Gannoun, Joseph Dewulf, Emile Van Schaftingen

**Affiliations:** ^1^ Metabolic Research Group de Duve Institute and UCLouvain Brussels Belgium; ^2^ Biochemical Genetics and Newborn Screening Laboratory, Department of Laboratory Medicine, Cliniques Universitaires Saint‐Luc, UCLouvain Brussels Belgium; ^3^ Institut des Maladies Rares, Cliniques universitaires Saint‐Luc, UCLouvain Brussels Belgium

## Abstract

Neutropenia in Glycogen Storage Disease Type Ib (GSDIb) and G6PC3 deficiency results from defects in metabolite repair, leading to the accumulation of 1,5‐anhydroglucitol‐6‐phosphate (1,5‐AG6P). Treatment currently relies on inhibitors of SGLT2, the renal sodium‐glucose co‐transporter, which indirectly enhances urinary excretion of 1,5‐anhydroglucitol (1,5‐AG), the precursor of the toxic 1,5‐AG6P that accumulates in neutrophils and is at the origin of these patients' neutropenia. In this context, a detailed understanding of the formation, intestinal absorption, renal reabsorption, and metabolism of 1,5‐AG is essential. Here, we review the current knowledge of these mechanisms, their role in the pathophysiology of 1,5‐AG6P–related neutropenia, and explore potential strategies to improve treatment outcomes.

## 1 Introduction

### 1.1 Metabolism and Repair: A Key to Sustaining Health

DNA repair is universally recognized as crucial, as it ensures the accurate transmission of genetic information from parent to daughter cells or organisms. However, the idea that metabolites also require repair is less widely acknowledged. Yet, the ancient origins of some identified repair mechanisms and the fact that defects in metabolite repair enzymes lead to diseases make it clear that metabolite repair is indeed important [1, 2, 3].

**FIGURE 1:**
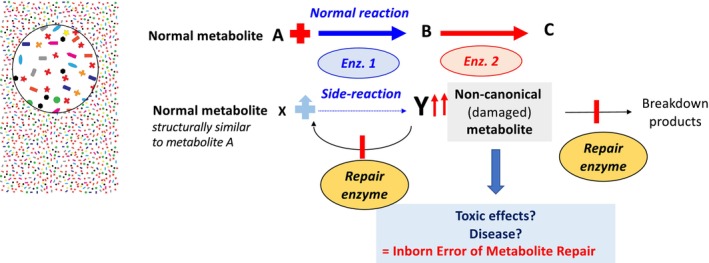
Illustration of the concept of metabolite repair. Enzymes involved in intermediary metabolism are not as specific as commonly assumed. While their primary function, such as the conversion of A to B by enzyme 1, is essential for established metabolic pathways, their imperfection and the crowded cellular environment can cause them to act on structurally similar metabolites (e.g., X). This results in side reactions and the formation of unintended products (e.g., Y), making metabolic errors unavoidable. These non‐canonical metabolites can disrupt metabolic homeostasis, for instance, by inhibiting enzyme activity, and must therefore be either degraded or converted back into normal metabolites. This metabolite clean‐up is carried out by metabolite repair enzymes. When these enzymes are deficient, non‐canonical metabolites accumulate within the cell, potentially exerting toxic effects and giving rise to inborn errors of metabolite repair.

Biochemistry textbooks describe enzymes of intermediary metabolism as highly specific for their substrates and the reactions they catalyze. However, this specificity is not absolute. As a result, many, if not all, enzymes act on structurally similar metabolites present in cells, albeit at rates 10^3^–10^6^ times lower than their primary physiological reactions. While these side reactions may seem negligible, the problem arises when the resulting abnormal metabolites are not suitable substrates for classical metabolic enzymes. Without specific metabolite repair enzymes to eliminate them, these abnormal compounds can accumulate to toxic levels (Figure 1).

Since L‐2‐hydroxyglutaric aciduria was recognized as the first inborn error of metabolite repair [4, 5, 6], various other inborn errors of metabolism (IEM) were shown to be the result of the accumulation of non‐canonical, abnormal, or damaged metabolites, resulting from side reactions of enzymes that catalyze reactions in the well‐known metabolic pathways [1, 2, 3, 7, 8]. Such non‐canonical metabolites can also arise from spontaneous reactions due to the inherent instability of certain molecules. For instance, NADH and, even more so, NADPH are unstable and can react spontaneously with water, leading to the formation of the hydrated forms NADHX and NADPHX [9, 10], which inhibit multiple dehydrogenases. Deficiency in the repair enzymes that eliminate these hydrated forms of the co‐factors [1, 11, 12] results in severe encephalopathies triggered by fever [13].

The challenge in uncovering metabolite repair mechanisms operating within cells suggests that probably many still unexplained metabolic diseases may result from defects in such processes. However, a major challenge in identifying the enzymes involved in such mechanisms lies in their activity on non‐classical and often low‐abundance metabolites. With this in mind, a defect in metabolite repair should come to mind when, despite thorough investigation, a clear connection between an enzyme's loss of function and the resulting pathophysiology remains elusive. In many such cases, the missing link may be the enzyme's still unrecognized role in metabolite repair. A perfect illustration of these considerations is the recent discovery by Linster et al. [14, 15] of the physiological role of CLYBL, a ubiquitous mammalian enzyme with an elusive role in the degradation of itaconate, but whose loss of function was mysteriously associated with vitamin B_12_ deficiency [16]. This metabolic puzzle was only solved when the function of CLYBL was also finally recognized as a metabolite repair enzyme.

**FIGURE 2:**
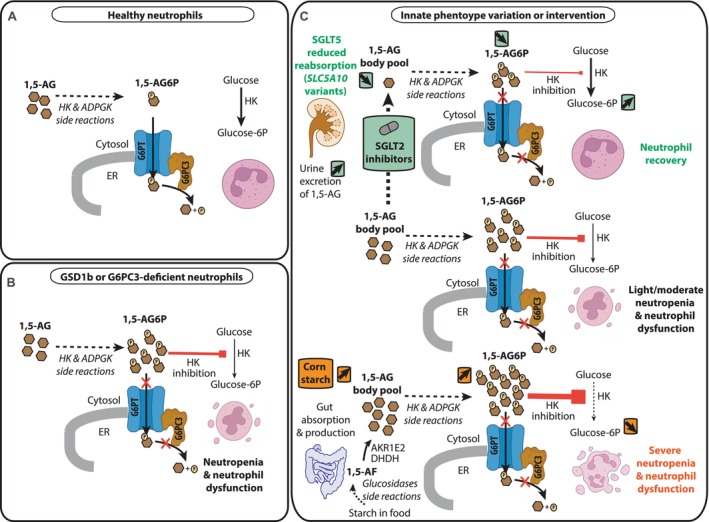
Illustration of 1,5‐anhydroglucitol metabolism in neutrophils, highlighting the impact of inactivating SGLT5 variants, starch‐rich diets, and SGLT2 inhibitor treatment in the context of GSD1b and G6PC3 deficiency. (A) In healthy neutrophils, circulating 1,5‐anhydroglucitol (1,5‐AG) enters the cells and is phosphorylated in the cytosol through side activities of glucose‐phosphorylating enzymes, such as low‐Km hexokinases and ADP‐dependent glucokinase. The resulting 1,5‐anhydroglucitol‐6‐phosphate (1,5‐AG6P) is transported into the endoplasmic reticulum via the G6PT transporter, where it is hydrolyzed by G6PC3 into inorganic phosphate and 1,5‐AG. (B) In neutrophils from patients with GSD1b or G6PC3 deficiency, 1,5‐AG6P accumulates due to impaired activity of either G6PT or G6PC3. Since 1,5‐AG6P is a potent inhibitor of all low‐Km hexokinases (soluble HK3 and mitochondria‐bound HK1, HK2), this leads to a depletion of glucose‐6‐phosphate and impairs essential metabolic pathways, including glycolysis, the pentose phosphate pathway, and protein glycosylation—ultimately resulting in neutrophil dysfunction and neutropenia. (C) Blood levels of 1,5‐AG are influenced by both diet and genetic factors. A starch‐rich diet, commonly followed by GSD1b patients, is expected to increase circulating 1,5‐AG levels, exacerbating neutropenia and complicating treatment. Conversely, individuals carrying loss‐of‐function variants in *SLC5A10*—the gene encoding the renal SGLT5 transporter specific for 1,5‐AG—exhibit reduced blood levels of 1,5‐AG. This observation underpins the rationale for developing specific SGLT5 inhibitors. 1,5‐AG, 1,5‐anhydroglucitol; 1,5‐AG6P, 1,5‐anhydroglucitol‐6‐phosphate; ADPGK, ADP‐dependent glucokinase; AKR1E2, aldo‐keto reductase family 1 member 2; DHDH, dimeric dihydrodiol dehydrogenase; ER, endoplasmic reticulum; G6PC3, Glucose‐6‐phosphatase catalytic subunit 3; G6PT, Glucose‐6‐phosphate transporter; Glucose‐6P, Glucose‐6‐phosphate; HK, hexokinase. Illustrations of the two proteins and the neutrophils have been downloaded from Biorender. The figure was made in Adobe Illustrator CS6.

Another remarkable example of a metabolite repair defect, at the center of this review article, is the genetically inherited neutropenia found in glycogen storage disease type Ib (GSDIb) and G6PC3 deficiency [17]. These two forms of neutropenia are particularly interesting because they cause a severe health problem that can be effectively managed with the repurposing of a widely available and relatively simple treatment. Additionally, in some cases, a modifier gene variant can reduce the severity of these forms of neutropenia, making treatment easier [18, 19] (Figure 2).

### 1.2 Understanding Inborn Errors of Metabolite Repair: Unlocking Innovative Treatment Strategies

When considering the complexity of the various mechanisms involved in metabolite damage, repair, and preemption that nature has evolved to prevent the accumulation of harmful by‐products, one may wonder whether we will ever succeed in treating these inborn errors of metabolite repair and truly help the affected patients. Yet, research into these defects has already opened several promising therapeutic approaches that are being explored:

1. *Increasing the concentration of the normal metabolite*: this strategy has been put in place in an attempt to help treat patients with a defect in NAD(P)HX repair by giving niacin as an NAD precursor. Available literature suggests mixed results, with several reports describing improvement in clinical status and a reversal of primary metabolic abnormalities [20, 21, 22] and one reporting an unfavorable outcome despite showing initial stabilization with a high dosage of niacin [23].
2. *Slowing the activity of the enzyme producing the abnormal metabolite*: This strategy aims at reducing the production of the non‐classical and harmful metabolite D‐2‐hydroxyglutarate (D‐2HG). D‐2HG is primarily formed as a side product of NADPH‐dependent isocitrate dehydrogenases (IDHs). Its accumulation is normally prevented by D‐2‐hydroxyglutarate dehydrogenase, a mitochondrial metabolite repair enzyme that oxidizes D‐2HG back to α‐ketoglutarate, an intermediate of the Krebs cycle [24, 25]. A deficiency of this enzyme leads to D‐2‐hydroxyglutaric aciduria type I [26]. In contrast, D‐2‐hydroxyglutaric aciduria type II is caused by mutations in the catalytic site of IDH2 that overwhelm the repair capacity of D‐2‐hydroxyglutarate dehydrogenase, resulting therefore in excessive D‐2HG production. In this case, treatment with enasidenib, a selective IDH2 inhibitor, has been shown to normalize D‐2HG levels in body fluids and improve clinical symptoms such as cardiomyopathy and seizures [27, 28].
3. *Reducing the quantity of the precursor of the abnormal metabolite*: this strategy, the focus of this review, was recently put in place to lower blood levels of 1,5‐AG, the precursor of the harmful abnormal metabolite, 1,5‐AG6P, that accumulates in neutrophils of GSDIb and G6PC3‐deficient patients. It consists of the repurposing of an SGLT2 inhibitor to promote the urinary excretion of 1,5‐AG (recently reviewed in [29]).

## 2 Origin of the Neutropenia in GSDIb and G6PC3‐Deficiency

### 2.1 Glycosylation Defects and Neutrophil Dysfunction

A glycosylation defect is found in neutrophils from both GSDIb and G6PC3 deficient patients and has been linked to enhanced apoptosis and ER stress [18, 30, 31]. This likely results from impaired glucose phosphorylation that presumably reduces the intracellular pools of UDP‐glucose and GDP‐mannose. Indeed, neutrophils in these patients exhibit hypoglycosylation of critical proteins like gp91(phox) (essential for NADPH oxidase activity) and truncated N‐ and O‐glycans. For example, G6PC3‐deficient neutrophils show reduced galactose and sialic acid residues, which may impair neutrophil function [31].

Yet, despite the above observation, the defect in glycosylation observed in neutrophils deficient in G6PC3 or G6PT is probably not the primary cause of neutrophil dysfunction, as most glycosylation disorders do not lead to neutropenia [32, 33].

### 2.2 Alternative Substrates for Metabolism in Neutrophils

Following the discovery of the glycosylation defect in neutrophils from GSD1b and G6PC3‐deficient patients, efforts to bypass hexokinase inhibition in neutrophils have been tried in GSD1b patients with galactose supplementation therapy, but the results are mixed [34]. Galactose treatment showed only marginal improvement, likely due to partial correction of glycosylation defects. Yet, the concentration of blood 1,5‐AG was not measured, and we therefore cannot exclude a weak inhibition of SGLT5, the 1,5‐AG renal transporter, by galactosuria [19] (see Section 6).

Another potential strategy could be the use of mannose supplementation to address the glycosylation defect in neutrophils. However, mannose is unlikely to exert its effect by supporting glycolysis or glycosylation, since its metabolism also depends on phosphorylation by hexokinase, which is strongly inhibited by 1,5‐AG6P in the neutrophils of these patients. Instead, mannose supplementation might act by inhibiting the renal transporter SGLT5 (see Section 6), thereby reducing renal reabsorption of 1,5‐AG and its concentration in blood [19]. Nevertheless, caution is warranted. Given the rapid cellular metabolism of mannose, the dose required to achieve urinary mannose concentrations high enough to inhibit SGLT5 and significantly lower blood 1,5‐AG may be far too high for safe human consumption. This is particularly concerning in the context of GSD1b, where patients already accumulate excessive G6P. Mannose supplementation could exacerbate this problem, as mannose is converted into mannose‐6‐phosphate, which can subsequently be converted into G6P through the sequential actions of phosphomannose isomerase (PMI) and phosphoglucose isomerase (PGI).

### 2.3 Inhibition of Hexokinase Activity by 1,5‐Anhydroglucitol‐6‐Phosphate

GSDIb results from a defect in G6PT, the transporter that enables glucose‐6‐phosphate (G6P) to move from the cytosol, where it is produced via gluconeogenesis and glycogen breakdown, into the endoplasmic reticulum (ER) of hepatocytes and kidney cells. In the ER, G6P is hydrolyzed by glucose‐6‐phosphatase (G6PC1), a transmembrane protein [35]. Metabolically, GSDIb closely resembles the classical form of GSDIa, which is caused by G6PC1 deficiency. Both conditions lead to lactic acidosis, hypoglycemia, glycogen accumulation in the liver and kidneys, and hyperuricemia. However, GSDIb has a distinguishing feature: it is almost invariably associated with neutropenia and neutrophil dysfunction.

For nearly 50 years, the mechanism linking G6PT deficiency to neutropenia remained a mystery. A major breakthrough came with the discovery of G6PC3, a phosphatase also present in the ER [36, 37]. Unlike G6PC1, which is expressed mainly in glucose‐producing tissues (liver, kidney, and intestinal mucosa), G6PC3 is ubiquitously distributed across tissues. In addition, while G6PC1 efficiently hydrolyzes G6P, G6PC3 has broader substrate specificity and, unlike it has often been stated, G6P is not one of its preferred substrates [17, 38]. Importantly, G6PC3 deficiency results in Severe Congenital Neutropenia type 4 (SNC4) [39], a type of neutropenia similar to that present in GSDIb [40]. This led to the hypothesis that both proteins (G6PT and G6PC3) work together to hydrolyze a phosphate ester, whose accumulation is toxic to neutrophils.

In 2019, the compound responsible for such toxicity was eventually identified as 1,5‐anhydroglucitol‐6‐phosphate (1,5‐AG6P), also called 1‐deoxyglucose‐6‐phosphate. It is structurally similar to G6P, with only one oxygen atom less on carbon 1, is well transported by G6PT into the ER, and is approximately 5 times more efficiently dephosphorylated by G6PC3 than G6P [17].

1,5‐AG6P is produced via phosphorylation of 1,5‐AG, a polyol present in blood, by low Km hexokinases (HK1, HK2, HK3, and most likely HKDC1), and ADP‐dependent glucokinase (ADPGK) [17, 41]. The catalytic efficiency of low‐Km hexokinases on 1,5‐AG is far below their catalytic efficiency on glucose (10 000 to 30 000‐fold lower), whereas, in comparison, ADPGK is significantly more active on 1,5‐AG (~1/20 of its activity on glucose) [17]. Consequently, in neutrophils, there are three key enzymes involved in the phosphorylation of 1,5‐AG: HK3, the most active hexokinase in neutrophils [42, 43], HK1, and ADPGK.

Neutrophils from G6PC3‐deficient and GSDIb patients accumulate 1,5‐AG6P to concentrations approaching 3 mM, which strongly inhibits low‐Km hexokinases such as HK3 and HK1. Consequently, glucose phosphorylation is reduced to less than 20% of its normal rate [17]. This inhibition is particularly problematic for mature neutrophils, which reduce their mitochondrial content during maturation in the bone marrow, specifically during their transition from the metamyelocyte to the band and segmented (mature) state neutrophils. During this process, mitochondrial number and activity decline significantly, and energy production shifts away from oxidative phosphorylation to glycolysis [44]. This allows for rapid energy production even in low‐oxygen environments typical of inflamed or bacteria‐infected tissues. As a result, neutrophils rely almost entirely on glucose phosphorylation for ATP production via glycolysis. This is essential for processes such as chemotaxis, endocytosis, and neutrophil extracellular traps formation (NETosis), as well as for NADPH generation in the oxidative pentose phosphate pathway, which is required for respiratory burst activity [45].

The accumulation of 1,5‐AG6P in neutrophils now provides an explanation for an observation reported nearly 30 years ago [46]: the phosphorylation of glucose was markedly reduced in neutrophils from patients with GSDIb and G6PC3 deficiency. Detailed analyses using low concentrations of glucose and glucose analogs showed that this reduced phosphorylation rate in intact cells was not due to impaired glucose uptake, but rather to impaired phosphorylation, despite normal hexokinase activity in cell‐free extracts [46]. Notably, both 1,5‐AG6P and G6P competitively inhibit low‐Km hexokinases with respect to ATP. The decrease in ATP concentration observed in neutrophils from GSDIb patients [47] and G6PC3‐knockout mice [48] may therefore enhance the inhibitory effect of 1,5‐AG6P. This aligns with observations made when treating patients, where even a modest reduction in blood 1,5‐AG levels can significantly improve neutrophil count and function.

### 2.4 Neutrophil Dysfunction in GSDIb and G6PC3 Deficiency

Neutrophils in these disorders are not only reduced in number (low neutrophil counts) but also functionally impaired [30, 49]. This appears to result from both a reduced production of mature neutrophils in the bone marrow and an increased susceptibility to apoptosis once in the circulation. Interestingly, treatment with granulocyte colony‐stimulating factor (G‐CSF) increases neutrophil counts but does not restore their normal function [49]. This likely explains why G‐CSF alone is often insufficient to prevent recurrent infections, whereas SGLT2 inhibitors—which, unlike G‐CSF, target the root of the problem and can restore neutrophil function without necessarily normalizing neutrophil counts—are more effective [18, 30].

Of note, the susceptibility to infections characterized by mouth ulcers, skin and perianal abscesses, and inflammatory bowel disease‐like symptoms, as is often described in the neutropenia present in GSD1b and G6PC3‐deficient patients, is also found in chronic granulomatous disease (CGD). In this inherited primary immunodeficiency, neutrophils are not numerically deficient, but they cannot effectively kill and phagocytose certain bacteria and fungi because they fail to generate reactive oxygen species due to defects in the NADPH oxidase complex [50, 51, 52]. This highlights the importance of functional defects in neutrophils to explain the symptoms of these forms of neutropenia, over just a mere reduction in neutrophil counts.

## 3 Formation of 1,5‐Anhydroglucitol: Can This Process Be Influenced or Controlled?

We next review the current knowledge regarding the formation of 1,5‐AG, a naturally occurring monosaccharide found in most foods and in human blood [53]. Its concentration in the body is regulated by a balance between dietary intake/intestinal absorption and renal reabsorption. Importantly, because 1,5‐AG Is the precursor of the toxic compound 1,5‐AG6P, which accumulates in neutrophils of patients with GSDIb and G6PC3 deficiency, it is crucial to understand the pathways involved in its production. Targeting one or more of these pathways may offer novel strategies to reduce 1,5‐AG levels in the body and thereby improve treatment outcomes for neutropenia in these patients.

### 3.1 Formation of 1,5‐Anhydrofructose in Microorganisms

Many polysaccharides are broken down via hydrolytic reactions, in which water attacks the glycosidic bond, producing aldoses. However, in some cases, the reaction does not use water as a substrate, and degradation occurs through an elimination reaction instead, producing a hexose with an additional double bond.

In glycogen and starch, degradation typically proceeds via phosphorylases or hydrolases, which respectively utilize phosphate or water to cleave glycosidic bonds, yielding glucose‐1‐phosphate or glucose. Certain microorganisms, such as fungi and red algae, also have glycoside lyases, notably α‐1,4‐glucan lyase, EC 4.2.2.13, [54], which catalyze an elimination reaction that forms a glucose derivative lacking a hydroxyl group at C1 and featuring a double bond between C1 and C2 (i.e., the enol form of 1,5‐anhydrofructose). This compound spontaneously tautomerizes to 1,5‐anhydrofructose [55, 56].

1,5‐Anhydrofructose can be further metabolized in fungi into other compounds, such as ascopyrone and microthecin [57, 58], which exhibit antibacterial activity. Alternatively, its keto group can be reduced to form 1,5‐anhydroglucitol (see below). If the microorganisms that produce 1,5‐anhydrofructose also have metabolic pathways to utilize it, this could represent a competitive strategy, allowing them to sequester carbon sources unavailable to others, which could be an explanation for developing such metabolic pathways.

All known glycoside lyases appear to belong to the GH31 (glycoside hydrolase family 31) protein family (GH31 lyases) [59]. Their mechanism involves forming a glucosyl‐enzyme intermediate, where the sugar binds to a strictly conserved aspartate residue (e.g., D553 in *G. lemanneiformis* GH31 lyase, PDB: 2 × 2I [60]), followed by an elimination reaction that produces the enol form of 1,5‐anhydrofructose and eventually the more stable keto form.

Importantly, 1,5‐anhydrofructose can also be formed, albeit as a minor side reaction, by glycoside hydrolases, also part of the GH31 protein family (Table 1; see also Section 3.4). These enzymes primarily catalyze hydrolysis (using thus water), producing glucose. However, in rare catalytic events, water is not used, and 1,5‐anhydrofructose is produced instead [64]. GH31 glycosidases, like their GH31 lyase counterparts, also form a glycosyl‐aspartate intermediate (e.g., via D443 in the N‐terminal domain of human maltase‐glucoamylase—MGAM) [66]. This intermediate is normally hydrolyzed due to catalytic residues that activate water and are absent in the lyase members of the GH31 protein family (GH31 lyases).

**TABLE 1:** Transporters and enzymes potentially influencing blood levels of 1,5‐anhydroglucitol based on Genome‐Wide Association Studies (GWAS).

Gene and protein encoded	Function	Tissular expression	Role related to 1,5‐AG	Link to [1,5‐AG]; references
Transporter				
*SLC5A10* (SGLT5)	Transport of: 1,5‐AG > mannose > fructose	Kidney cortex – apical membrane proximal convoluted tubule	Renal reabsorption of 1,5‐AG into the bloodstream preventing urinary excretion	Genetic link to 1,5‐AG levels [61, 62, 63]
*SLC5A1* (SGLT1)	Transport of: glucose > galactose >> 1,5‐AG	Small intestine – apical membrane of enterocytes; Kidney cortex ‐ apical membrane proximal convoluted tubule	Intestinal absorption of 1,5‐AG	Genetic link to 1,5‐AG levels [61, 62, 63]
*EFNA/SLC50A1*	Transport of: glucose?	Ubiquitous	Non energy driven transport of 1,5‐AG in out of cells?	Genetic link to 1,5‐AG levels [61]
β‐GLYCOSIDASES				
*MCM6/LCT* (Lactase/Phlorizin hydrolase)	Hydrolysis of: β‐galactosides; β‐glucosides	Small intestine – brush border of enterocytes	Enzyme side‐activity forms 1,5‐AF?	Genetic link to 1,5‐AG levels [61, 62, 63]
α‐GLYCOSIDASES				
*MGAM* (two catalytic domains: Maltase/Glucoamylase)	Hydrolysis of: maltose; maltotriose; α‐1,4‐glycosidic bonds in starch‐derived oligosaccharides	Small intestine – brush border of enterocytes	Enzyme side‐activity forms 1,5‐AF?	Genetic link to 1,5‐AG levels [61, 62, 63]
*MGAM2* (Maltase‐Glucoamylase 2) putative protein‐coding gene in humans; pseudogene?	Inactive protein or altered enzymatic specificity compared to MGAM? Lacks one active site residue.	Salivary glands Small intestine – brush border of enterocytes. 100‐fold lower expression compared to MGAM	Enzyme side‐activity forms 1,5‐AF?	Genetic link to 1,5‐AG levels [61, 62]
*SI* (two catalytic domains: Sucrase/Isomaltase)	Hydrolysis of: sucrose; isomaltose	Small intestine – brush border of enterocytes	Enzyme side‐activity forms 1,5‐AF?	Genetic link to 1,5‐AG levels [61, 63]
*GANAB* (Catalytic subunit of neutral α‐glucosidase II)	Protein processing in the ER (glycoprotein maturation): sequential removal of the two innermost α‐1,3‐glucosides on newly synthesized glycoproteins	Ubiquitous; Endoplasmic reticulum	Low in vitro production of 1,5‐AF	In vitro generation of 1,5‐AF [64]
*GANC* (cytosolic α‐glucosidase)	Hydrolysis of: α‐1,4‐linked glucoside from glycogen	Ubiquitous; Cytosol	Low in vitro production of 1,5‐AF	In vitro generation of 1,5‐AF [65]

*Note:* Three independent GWAS [61, 62, 63] have associated blood levels of 1,5‐AG with various genes encoding transporters involved in its intestinal and renal reabsorption. Additionally, these studies identified enzymes (α‐ and β‐glycosidases) involved in carbohydrate digestion with potential side activities generating 1,5‐anhydrofructose. Two of the α‐glucosidases listed were not identified through GWAS but have independently been shown to produce small amounts of 1,5‐anhydrofructose in vitro.

Abbreviation: 1,5‐AG, 1,5‐anhydroglucitol, 1,5‐AF, 1,5‐anhydrofructose.

In GH31 *lyases*, the elimination reaction requires a base catalyst to abstract a proton (H^+^), a role apparently played by the catalytic residue D553, which is assisted by the hydrogen bond it forms with residue N459. In GH31 *glycosidases*, N459 is replaced by isoleucine, which significantly impairs the elimination reaction. Nevertheless, occasional elimination does occur. For instance, α‐glucosidase from *Aspergillus niger*, when incubated with glycogen, produces 1 molecule of 1,5‐anhydrofructose for every ~10,000 glucose molecules in 10 min [67]. Though this yield seems negligible, it aligns with daily dietary levels of 1,5‐AG, about 5 to 10 mg [53], compared to ~100 to 200 g of starch. Thus, following its reduction to 1,5‐AG, 1,5‐anhydrofructose produced as a side product by GH31 glycosidases likely accounts for most of the 1,5‐AG found in food, more so than GH31 lyase‐derived pathways.

### 3.2 Potential Mechanism of Formation of 1,5‐Anhydrofructose in Vertebrates

Given the conservation of the catalytic residues in GH31 glycosidases, the elimination side‐reaction producing 1,5‐anhydrofructose is likely present in many glycosidases, though its frequency may vary between enzymes. In mammals, this has been demonstrated for glucosidase II (GANAB), which processes glycosides in the context of N‐glycosylation in the ER [64] and for a cytosolic glycogen‐hydrolyzing enzyme [65], presumably GNAC. While 1,5‐anhydrofructose formation has been observed in both cases, the relative rates of hydrolysis versus elimination remain unquantified (Table 1).

Due to the high conservation of catalytic residues, it is likely that other human GH31 enzymes also produce small amounts of 1,5‐anhydrofructose (Table 1). Supporting this, genome‐wide association (GWAS) studies identified several genes encoding glucosidases involved in carbohydrate digestion that are associated with the level of 1,5‐AG in blood [61, 62]. These include sucrase‐isomaltase (*SI*), and the two maltase‐glucoamylases (*MGAM* and *MGAM2*), suggesting that these enzymes contribute significantly to the intestinal production of 1,5‐anhydrofructose, and consequently 1,5‐AG, during carbohydrate digestion. As a result, starch‐rich diets could lead to elevated blood levels of 1,5‐AG, which might explain why patients with GSDIb tend to have higher baseline 1,5‐AG levels and respond less effectively to SGLT2 inhibitor therapy compared to those with G6PC3 deficiency, making the neutropenia in GSDIb patients more challenging to treat. Intriguingly, blood 1,5‐AG levels are also linked to sequence variations near the gene *LCT*, encoding lactase [61], indicating that lactase activity or most probably its associated β‐glucosidase activity could also produce small amounts of 1,5‐anhydrofructose from β‐glucosides.

### 3.3 Reduction of 1,5‐Anhydrofructose to 1,5‐Anhydroglucitol

1,5‐Anhydrofructose is readily reduced to 1,5‐AG. This is demonstrated by studies using radiolabelled precursors in rats: after oral administration, 48% of absorbed 1,5‐anhydrofructose was excreted in urine as 1,5‐AG within 6 h [58]. Similarly, oral administration in humans and minipigs results in 1,5‐AG appearing in blood and urine, while 1,5‐anhydrofructose remains undetectable [68].

An enzyme that reduces 1,5‐anhydrofructose to 1,5‐AG was purified from pork liver and its sequence was established [69, 70]. The human homolog is AKR1E2 (70% identity), while the mouse homolog is AKR1E1 (75%). However, more recent findings suggest that dimeric dihydrodiol dehydrogenase (DHDH) is a more efficient reductase of 1,5‐anhydrofructose in humans [71]. DHDH is most highly expressed in proximal enterocytes and proximal tubular cells, consistent with the intestinal absorption and renal reuptake of 1,5‐AG. As discussed above, it may therefore be the enzyme that was found to reduce 1,5‐anhydrofructose when administered orally to humans and other vertebrates [68].

*Escherichia coli* has also been shown to synthesize both 1,5‐AG and 1,5‐anhydrofructose via an undefined pathway [72]. Finally, the presence of 1,5‐AG in numerous food items [73] suggests the existence of 1,5‐anhydrofructose reductases in plants, though no such enzyme has yet been identified or characterized in these species.

### 3.4 Presence of 1,5‐Anhydroglucitol in Food

Analyses of various food items indicate that 1,5‐AG is present in virtually all of them, making the formulation of a 1,5‐AG‐free diet, which could be ideal to resolve neutropenia in GSDIb or G6PC3‐deficient patients, nearly impossible. A study by Yamanouchi et al. [53] found that the daily urinary excretion of 1,5‐AG by Japanese volunteers slightly exceeded their estimated intake. This suggests that the majority of 1,5‐AG in the body originates from dietary sources, with only a small portion, approximately 10%, produced endogenously.

The average daily dietary intake of 1,5‐AG was estimated to be around 4.38 ± 0.85 mg, consistent with food analysis data showing an average of 0.22 mg of 1,5‐AG per 100 kcal in a typical Japanese diet [53]. In a study involving healthy subjects (*n* = 36), the average daily urinary excretion of 1,5‐AG was approximately 4.76 ± 0.89 mg, while fecal excretion was negligible. In addition, observations in non‐diabetic individuals (*n* = 6) who were not orally fed, but instead received a 1,5‐AG‐free intravenous hyperalimentation over 14 days [53], indicated an endogenous synthesis rate of about 0.4 mg/day. Taken together, these findings support the following conclusions: (1) 1,5‐AG originates primarily from dietary sources (see Sections 3.1 and 3.2 on intestinal starch‐derived production) and is efficiently absorbed in the intestine; (2) 1,5‐AG undergoes minimal degradation or metabolic transformation within the body; (3) a physiological balance is maintained between dietary intake, a modest but steady endogenous synthesis, and urinary excretion.

## 4 Absorption of 1,5‐Anhydroglucitol

### 4.1 Intestinal Absorption

It is well established that 1,5‐AG is readily absorbed in the intestinal tract. In rats, serum levels of 1,5‐AG increased within 30 min following oral administration of 600 mg of this polyol, and nearly 100% of the compound was excreted in the urine within 24 h [74]. This dosage is extremely high, approximately 60‐fold greater than the average daily human intake, and exceeds the reabsorption capacity of the transporter of 1,5‐AG (SGLT5) in the renal proximal tubule. Nevertheless, the findings clearly demonstrate efficient intestinal absorption of 1,5‐AG.

Similarly, in humans, serum 1,5‐AG peaked 30 min after oral administration of 20 g (approximately 2000 times the typical daily intake) and gradually declined over the next 3 h. Approximately 60% of the ingested dose was excreted in the urine within 9 h. Notably, hydrogen excretion was minimal in both rats and humans 24 h post‐administration, indicating extensive intestinal absorption and minimal microbial fermentation of 1,5‐AG in the gut [74].

### 4.2 Transporters Possibly Involved in Intestinal Absorption of 1,5‐Anhydroglucitol

As discussed above, 1,5‐AG is clearly well transported through the intestinal barrier, but there is no precise information on both the apical and the basolateral transporter(s) involved in this process. Since 1,5‐AG resembles glucose, it is likely that transporters that have been established as intestinal glucose transporters, could contribute to the intestinal absorption of 1,5‐AG.

A first likely candidate for the apical transport is SGLT1, the sodium‐glucose co‐transporter 1, encoded by *SLC5A1*. It transports D‐glucose and D‐galactose into cells, using the sodium gradient as the driving force [75] and is predominantly expressed in the apical membrane of epithelial cells in the small intestine, especially from the duodenum to the ileum, with the highest expression in the proximal segments (duodenum and jejunum). Data obtained with human SGLT1 expressed in xenopus oocytes indicates that it also transports 1,5‐AG (1‐deoxyglucose) with a Km of 10 mM compared to 0.5 mM for glucose [76]. Consequently, SGLT1 is likely to contribute to 1,5‐AG intestinal reabsorption, although with approximately 20‐fold lower affinity for 1,5‐AG compared to glucose. In addition, the association of SGLT1 with 1,5‐AG intestinal absorption is supported by GWAS, indicating a linkage to blood levels of 1,5‐AG in two separate studies [61, 63].

SGLT4/SLC5A9, is probably not a major contributor. Although mainly expressed in the small intestine it was recently shown to have considerably lower affinity for 1,5‐AG than for mannose [19], which likely makes it an intestinal mannose transporter.

As for the transport at the luminal membrane, the exit of 1,5‐AG from the enterocyte into the circulation likely relies on transporters shared with glucose due to structural similarities. Consequently, the basolateral transport for 1,5‐AG in the intestine may involve SWEET1, a sugar transporter broadly expressed (encoded by *SLC50A1*) and mediating glucose efflux across cellular membranes [77]. Remarkably, GWAS have linked *SLC50A1* to blood concentration of 1,5‐AG [61], suggesting a potential role in basolateral transport. However, this transporter remains poorly characterized in humans, and further research is needed to elucidate its precise function and involvement in this pathway.

### 4.3 Absorption of 1,5‐Anhydrofructose

Absorption of 1,5‐anhydrofructose in the intestine also appears to be highly efficient [68]. In its hydrated form, 1,5‐anhydrofructose has two hydroxyl groups on C2 instead of a ketone, making it structurally similar to glucose, mannose, and fructose (in their pyranose forms). This structural similarity suggests 1,5‐anhydrofructose may be transported by GLUT5, which is expressed in enterocytes. Specifically, this transporter is found at high levels in the apical membrane of intestinal epithelial cells in the small intestine, where it facilitates the uptake of fructose from the intestinal lumen into the cell (reviewed in [78]). The observation made by Ijiri and colleagues [68] when they studied the metabolism of 1,5‐anhydrofructose to 1,5‐AG, showing that 1,5‐anhydrofructose was only detected in blood after intravenous (but not oral) administration, suggests that once 1,5‐anhydrofructose enters the enterocyte by GLUT5 (or another glucose transporter), it is immediately reduced to 1,5‐AG by one of the dehydrogenases discussed above (see Section 3.3) to finally end up in the blood stream as 1,5‐AG (see Section 4.1).

### 4.4 Transport From the Blood Stream Across the Plasma Membrane Into Other Cell Types: The 1,5‐Anhydroglucitol Body Pool

Cellular uptake of 1,5‐AG was shown to occur competitively with glucose in various human and mouse cell lines, mouse primary hepatocytes and human and mouse white blood cells [17, 18, 30, 79, 80, 81]. These observations imply the involvement of facilitative glucose transporters such as GLUT1, GLUT2 and also GLUT3, to allow for 1,5‐AG to be transported dynamically across cell membranes, yet further studies are needed to identify the specific passive transporter(s) for 1,5‐AG. In contrast, 1,5‐anhydrofructose appears to be transported efficiently without interference from glucose (Veiga‐da‐Cunha, unpublished observation), supporting GLUT5 as a likely transporter in both intestinal and peripheral tissues (see Section 4.3).

## 5 Metabolism of 1,5‐Anhydroglucitol

### 5.1 In Bacteria

Significant progress in our understanding of 1,5‐AG metabolism in the gut microbiome has been recently made by Ma et al. [82]. These researchers identified anaerobic pathways for the breakdown of 1,5‐AG and 1,5‐anhydromannitol in bacteria present in the intestine. Both pathways begin with phosphorylation of the polyol to its 6‐phosphate form via a phosphotransferase system (PTS). The key enzymes involved in these pathways (YbiW and PflD in *E. coli* ) cleave 1,5‐AG6P and 1,5‐anhydromanitol‐6‐phosphate (1,5‐AM6P) respectively into 1‐deoxyfructose‐6‐phosphate, which is then further metabolized. This reaction requires a glycyl radical, rendering it highly oxygen‐sensitive and thus functional only under strictly anaerobic conditions.

These metabolic pathways enable *E. coli* and many other human gut bacteria to grow on 1,5‐AG [82], but most likely only under strict anaerobic conditions. In the human intestine, such low‐oxygen (hypoxic) environments are primarily found in the colon [83, 84], whereas 1,5‐AG is probably produced and absorbed in the more oxygen‐rich proximal regions of the small intestine. As a result, stimulating bacterial metabolism of 1,5‐AG to reduce its intestinal absorption is unlikely to be effective. This suggests that efforts to develop a probiotic to lower intestinal 1,5‐AG levels, and thereby reduce its absorption, are unlikely to succeed, unless an enzyme capable of metabolizing 1,5‐AG under aerobic conditions can be identified.

In contrast, aerobic soil bacteria from the *Rhizobiaceae* family are capable of metabolizing 1,5‐anhydrofructose via a different route. The metabolic pathway begins by a specific reductase that converts 1,5‐anhydrofructose to 1,5‐anhydromannitol. This is followed by hydroxylation at carbon 1 by a cytochrome P450‐family‐like enzyme (still unidentified), yielding mannose [85]. This pathway was elucidated after isolating *Sinorhizobium morelense* S‐30.7.5 using 1,5‐anhydrofructose as the sole carbon source. Additionally, the fungus *Aspergillus niger* can grow on 1,5‐anhydrofructose, whereas *Saccharomyces cerevisiae* cannot [66], but an analogous oxidative pathway metabolizing 1,5‐AG has not been described.

### 5.2 In Mammals

From the limited published work on this subject, there is no evidence of significant 1,5‐AG catabolism in mammals, despite it being one of the main polyols found in the human body [86, 87]. It is actively absorbed in the intestine and actively transported during the renal reabsorption, yet it is not catabolized in cells. Minimal oxidation was observed following the injection of radiolabeled 1,5‐AG or 1,5‐anhydrofructose into mice, and it remains unclear whether this results from actual metabolism of 1,5‐AG or from the metabolism of impurities in the preparation [68]. It is found in all mammalian tissues, with a phosphorylated form (1,5‐AG6P) reported in rats that comprised up to 24% of total 1,5‐AG in the spleen [88].

In cultured cells, radiolabeled 1,5‐AG enters rapidly and is partially phosphorylated. Saturation curves suggest the presence of at least two distinct components. Glucose inhibits phosphorylation down to ~15%, further supporting the existence of multiple pathways [88]. In these experiments, the resulting phosphorylated pool was described as unstable, likely because it is subject to rapid dephosphorylation. We now know that this is the result of the collaboration between G6PT and the ER resident phosphatase G6PC3 that act together to prevent the accumulation of 1,5‐AG6P [17].

Hexokinases 1, 2, and 3 phosphorylate 1,5‐AG in vitro, but their catalytic efficiencies are about 10 000 times lower than for glucose [17]. ADP‐dependent glucokinase (ADPGK), an ER bound enzyme with an unclear physiological role, also phosphorylates 1,5‐AG, but with a catalytic efficiency roughly 20 times lower than for glucose. While ADPGK has lower activity than hexokinases in tissues, both enzyme types may contribute to 1,5‐AG phosphorylation in different tissue‐specific proportions, possibly explaining the dual‐component pattern observed by Mizuno et al. [88].

1,5‐AG6P is a good substrate for G6PC3 and a much poorer one for the classical glucose‐6‐phosphatase, G6PC1. Studies in ER vesicles (microsomes) show that its hydrolysis is inhibited by a specific inhibitor of G6PT. Accumulation of 1,5‐AG6P in cells lacking functional G6PC3 or G6PT/SLC37A4 indicated that these proteins help eliminate this compound, supporting the view that 1,5‐AG6P serves no physiological function and may even be harmful [17]. Notably, 1,5‐AG6P strongly inhibits hexokinase [17, 89]. This inhibition occurs via binding to the G6P regulatory site, which is distinct from the catalytic glucose‐binding site. This explains the discrepancy between substrate and inhibitor specificities: glucose, mannose, and 2‐deoxyglucose are excellent substrates, but only G6P is a strong inhibitor [41, 89, 90]. Conversely, although 1,5‐AG is a poor substrate, its phosphate form is a potent inhibitor [17].

## 6 Urinary Elimination of 1,5‐Anhydroglucitol: A Key Target in the Treatment of Neutropenia

SGLT1 and SGLT2 are the Na^+^‐dependent glucose transporters responsible for most of the glucose absorption in the intestinal mucosa and the proximal renal tubule, respectively. SGLT4 and SGLT5 are sodium‐dependent transporters of mannose and fructose, expressed in intestinal and kidney proximal tubular cells [91].

Shortly after the discovery of 1,5‐AG in body fluids, it became evident that this compound is efficiently retained in the body via a Na^+^‐dependent transporter in the renal tubule. This transporter also acts on mannose, fructose, and the non‐physiological polyol 1,5‐anhydromannitol [19, 92, 93]. GWAS [61, 63, 94] and our own work [18] identified mutations in SGLT5 (*SLC5A10*) as being associated with lower blood levels of 1,5‐AG. We subsequently demonstrated in cell models that SGLT5 is a highly effective transporter of 1,5‐AG (Km = 160 μM), showing 3‐ and 6‐fold higher affinities for 1,5‐AG compared to mannose and fructose, respectively, and an even greater (10‐fold higher) affinity for 1,5‐anhydromannitol. SGLT5 also shows some affinity for glucose, but its transport activity is half‐maximally inhibited at a glucose concentration close to 5 mM [19].

When treating neutropenia in patients with GSD1b or G6PC3 deficiency, it is important to monitor their response to SGLT2 therapy. If a patient shows an especially strong response, both through increased neutrophil counts and reduced blood levels of 1,5‐AG, this may indicate the presence of a mutation in *SLC5A10*, which occurs in the heterozygous state in approximately 2% of the population [19]. Of note, inactivating mutations in SGLT5 cause in the heterozygous state an approximate 50% decrease in 1,5‐AG level in blood, indicating haploinsufficiency. In such cases, sequencing of *SLC5A10* is recommended. Confirmation of an inactivating variant would suggest reduced SGLT5 activity and increased urinary excretion of 1,5‐AG, thereby making neutropenia in these patients easier to manage [18].

Despite transporting 1,5‐AG, SGLT5 serves two key physiological functions in the kidney: (1) recovery of mannose, vital for protein glycosylation, and (2) recovery of fructose, an important dietary sugar. Additionally, reabsorption of fructose and mannose from the urinary filtrate may be beneficial to limit infections of the urinary tract [19, 93]. It is likely that evolution favored a transporter distinct from the glucose transporters SGLT1 and SGLT2 to avoid strong competitive inhibition. Since these two transporters are highly efficient glucose transporters, they are ill‐suited to recover other sugars from the glomerular filtrate. As a result, SGLT5, which is expressed in the renal proximal tubule further along from where SGLT2 and SGLT1 are expressed, is responsible for the reabsorption of fructose, mannose, and 1,5‐AG. Because of its structural similarity to glucose, 1,5‐AG is mistakenly and slowly phosphorylated by hexokinases and ADPGK to form 1,5‐AG6P, a potent inhibitor of hexokinases that is particularly toxic for neutrophils. To counteract this, nature has therefore evolved a repair system involving G6PT and G6PC3, which eliminates 1,5‐AG6P from the cells [17, 94].

Importantly, SGLT5's partial affinity for glucose results in reduced reuptake of 1,5‐AG under hyperglycemic conditions [29, 95]. This explains why blood levels of 1,5‐AG drop in diabetes, making it a reliable biomarker for glycemic control [73, 96, 97, 98]. Similarly, SGLT2 inhibitors (gliflozins), used in diabetes therapy, induce 1,5‐AG loss by indirectly inhibiting SGLT5 through glucosuria, resulting in a significant decrease in blood 1,5‐AG levels [99].

Based both on these observations and on the discovery of the key role that 1,5‐AG plays in the neutropenia of GSDIb and G6PC3‐deficient patients, it is not surprising that the therapeutic approach based on the usage of SGLT2 inhibitors is effective. Indeed, lowering intracellular 1,5‐AG6P levels in neutrophils improves their function, reduces infection rates, and often resolves inflammatory bowel disease (IBD) (recently reviewed in [29]). This is associated with an improvement in the intestinal absorption, which often leads to weight gain and resolves anemia in the most affected patients [100, 101, 102].

However, not all patients respond equally well. In particular, individuals with GSDIb, who tend to have higher baseline levels of 1,5‐AG in the blood, often do not achieve sufficient reductions in 1,5‐AG through urinary excretion alone to fully control neutropenia. This highlights the need for a deeper understanding of the factors that regulate 1,5‐AG concentrations in the body.

## 7 Concluding Remarks and Perspectives on Improving Treatment Strategies

Maintaining low concentrations of 1,5‐AG appears to be the most effective approach to treating neutropenia in patients with GSDIb and G6PC3 deficiency. While SGLT2 inhibitors help achieve this, their mechanism is indirect: by increasing glucose concentrations in the renal proximal tubule, they inhibit SGLT5, the transporter responsible for 1,5‐AG renal reabsorption. In this context, a specific SGLT5 inhibitor would likely be much more effective, with the added benefit of reducing the risk of hypoglycemia, particularly in patients with GSDIb.

Such inhibitors would probably differ structurally from gliflozins by featuring a sugar moiety with a hydroxyl group in the L‐orientation (as found in mannose and 1,5‐anhydromannitol), rather than the D‐orientation seen in glucose, phlorizin, and all currently known gliflozins. However, while this might represent an ideal therapeutic option, developing a specific SGLT5 inhibitor would be time consuming and expensive.

A more realistic and immediately actionable alternative may be to repurpose a gliflozin that inhibits both SGLT2 and SGLT5 with sufficient affinity. Direct inhibition of SGLT5 would complement the indirect effect mediated by elevated luminal glucose levels following SGLT2 inhibition. Remogliflozin is a particularly promising candidate in this regard, as it exhibits only a 16‐fold lower affinity for SGLT5 than for SGLT2 [103]. Yet, this gliflozin is only available in India [104], is less stable (it is given as a pro‐drug; remogliflozin‐etabonate) and has a shorter half‐life requiring a higher dosage to be given to patients [104, 105].

One other important question to address is why the neutropenia of some patients (particularly GSDIb ones) responds less effectively to the treatment with SGLT2 inhibitors. A possible contributing factor is the generation of 1,5‐AG (via 1,5‐anhydrofructose) from the digestion of uncooked cornstarch and maltodextrin, which are commonly administered in large amounts to GSDIb patients (see Figure 2C). Appropriate studies need to be designed to evaluate the importance of adjusting the level of these indispensable supplements to no more than what is strictly needed.

Finally, further investigation is needed to determine whether the accumulation of 1,5‐AG6P in blood cells other than neutrophils, such as lymphocytes and platelets, also impairs their function by inhibiting glycolysis. Occasional reports have indicated an increased risk of autoimmune disorders, mild lymphopenia, and immune dysfunction in patients with GSDIb (but not GSDIa) and G6PC3 deficiency [106, 107, 108, 109]. These abnormalities have been linked to a reduced ability of T cells to initiate glycolysis upon T‐cell receptor (TCR) stimulation [110]. Although it remains unproven whether these defects are directly caused by 1,5‐AG6P accumulation, if a link is established, SGLT2 inhibitor therapy could offer broader therapeutic benefits by addressing these additional immune impairments as well.

## Ethics Statement

The authors have nothing to report.

## Conflicts of Interest

The authors declare no conflicts of interest.

## Data Availability

Data sharing not applicable to this article as no datasets were generated or analyzed during the current study.
